# Hematoma Auris

**Published:** 1890-11

**Authors:** B. H. Grove

**Affiliations:** Surgeon to the Erie County Eye, Ear, and Throat Infirmary; 334 Pearl Street


### HEMATOMA AURIS.[1]

By B. H. GROVE, M. D.,

Surgeon to the Erie County Eye, Ear, and Throat Infirmary.

THIS affection is commonly known as the blood tumor of the ear, or the asylum ear. Its medical synonyms are perichondritis, or chondromalacosis auris. These terms are not, however, altogether applicable, and hence have been the source of considerable confusion.

1. Read before the Buffalo Pathological Society, September 19, 1890.

Some sixteen or eighteen cases of hematoma auris have come under my observation. The larger portion were among the mentally insane.

I am under obligations to Drs. Hurd and Bryant, of the Buffalo State Hospital, and Dr. Parker, of the Erie County Insane Asylum, as well as Dr. Meidenbauer, for the opportunity of witnessing lately some acute cases of hematoma auris, as well as some of the conditions following such attacks.

Hematoma auris is characterized by congestion and heat in the auricle, and a rapid effusion of blood or some of its constituents between the cartilage of the auricle and its perichondrium, or into the substance of the cartilage itself.

The tumor, in the course of a few hours or days, becomes the size of a bean or a small hen's egg. It is always situated on the anterior or concave side of the auricle. The auricle may retain its normal color, but more often it takes on a purplish hue. The tumor may pass through a low grade of inflammation.

It is in a mild degree hot and dense to the touch, and often fluctuation is barely perceptible. Its manipulation causes the patient but little pain.

All portions of the auricle, except the lobule, contain cartilage. Cartilage is also a partial constituent of about one-half the length of the external auditory canal. Naturally enough, then, the lobule is never involved. This is likewise the case with the tragus, for some unexplainable reason, although it contains cartilage. The patient generally complains of some burning pain, with a feeling of weight and distention in the new growth. The earliest manifestations of the disease are rapid, but after the tumor has become fully formed it may remain apparently in *statu quo* for days or weeks.

Finally, it may rupture spontaneously, which is the most general method of its disappearance. When this is not the case, the blood may become coagulated and absorbed. Whichever course is pursued, there nearly always follows a permanent and characteristic deformity of the auricle, which is more or less marked. When the tumor has reached its full size, it has a peculiar doughy feeling, which once experienced will be remembered. Its occurrence is generally unilateral, although in the insane it may be ambilateral. It is not uncommon, however, to find hematoma auris affecting each ear of the insane, with an interval of a few months to a few years. A little before, or at the time of the development of this blood tumor, there is often noticed, with the insane at least, during cerebral excitement, a congested appearance of the face, eyes and ears.

About fifty years ago, Dr. Henry Bird described six cases of hematoma coming under his observation. He noted the intimate relationship of the disease to insanity, but he could not account for its origin, having never traced it to traumatism. Since then various German, French, and American writers have contributed largely to this interesting subject.

The aurists, Gruber, Hartmann, Politzer, Buck, Roosa, and Pomeroy, have each mentioned a few typical cases among the mentally sane.

However, Virchow, in his work on tumors ; E. R. Hun, in the *American Journal of Insanity* for 1870, and Samuel Sexton, in the *Medical Record* of 1884, are the authors who, according to my opinion, have presented us with the most complete and satisfactory description of this interesting affection. In this paper, therefore, I shall draw largely from their experience in connection with my own limited observations.

We must not forget to mention later on, however, the instructive experiments of Dr. Brown-Séquard in regard to this subject.

It will be noticed, after comparing the literature on this affection, that there exists a great discrepancy in the conclusions arrived at by the various observers. This applies especially to the etiology and treatment, and even in a measure to the pathology. One author contends that hematoma occurs more often in the left than the right ear ; that males, by a large majority, are more often affected than females ; that never, or hardly ever, is it present with the sane ; that it is idiopathic, depending on pathological conditions of the brain, and is incapable of being produced by violence alone ; on the other hand, that, as a rule, violence, or traumatism of some sort, is the cause, both in the sane and insane.

Dr. Hun gives the history of twenty-four cases of hematoma auris in the insane. Of this number twenty-three occurred in males. The form of insanity was general paresis in eight, melancholia in six, acute mania in four, and dementia in two.

It will be observed from this classification that when hematoma auris occurs in the insane the symptom is to be considered unfavorable, because it suggests an incurable form of brain disease.

Dr. Sexton asserts that out of 1,300 female insane patients, sixteen cases of hematoma auris were found, or about 1.14 per cent.; and out of 1,200 male insane patients, fourteen cases, or about 1.10 per cent. On the other hand, out of nine cases among the mentally sane examined by the same author, all were males except one.

About fifteen cases among the mentally sane, besides the nine mentioned above, have been reported by American authors.

Of course, this must be but a small portion of actual cases. Two such cases have, not very long ago, come under my own observation, which have not been reported. Moreover, these were idiopathic in origin. The causes then, you notice, of hematoma auris among the sane are idiopathic as well as traumatic. With the insane, even, the origin may be traumatic, as well as dependent upon cerebral congestion and reflex irritation of the nerve centers, through the emotions. According to all authors there is always a tendency in general paresis to an unusual determination of blood to the head, and it is supposed that the blood-vessels of the ears become so dilated as to make the effusion possible. The other factor causing hematoma auris, namely, centripital irritation of the sympathetic, is present, especially with the insane, because they are subject to excessive attacks of excitement, while their emotions are but little under their control.

Dr. Hun is so strongly of the opinion that the idiopathic othematoma are symptoms of insanity, that he would consider it policy for any person having such a tumor, even if sane, to be carefully observed as to cerebral symptoms.

On the other hand, Dr. Sexton is convinced, from his investigations, that hematoma auris is nearly always caused, both in the mentally sane and insane, by some external means. From two cases under my own observation some time ago, I know hematoma auris can occur idiopathically in the mentally sane. Under treatment one case resulted in a deformed auricle, while the auricle of the second patient became virtually normal.

With regard to the etiology, the experiments of Dr. Brown-Séquard, as witnessed and described by Dr. Roosa, are interesting and instructive.

Dr. Brown-Séquard has found that sections of the restiform bodies, or largest columns of the medulla oblongata in animals (guinea pigs), will produce a hemorrhage beneath the skin of the auricle in from twelve to twenty-four hours. This hemorrhage is soon followed by gangrene of the part. The hemorrhage usually occurs in the fossa helicis of the auricle, and in the ear corresponding to the same side of the section.

Dr. Brown-Séquard also states that section of the sciatic nerve, by reflex action upon the medulla, would produce the same result. He believes that disease of the base of the brain, which is, however, not always attended by insanity, is the cause of hematoma auris. He thinks, moreover, that gangrene does not occur in man simply because the tissues of the ear are more capable of resisting the pressure than in the lower order of creation.

The pathology of hematoma auris has given rise to extensive discussion. The views are various, and often quite contradictory. Hun describes it more especially as an effusion of blood or serum between the cartilage and perichondrium — the cavity being of perfectly smooth surfaces,— and that the lesion is peculiar to the insane.

Virchow, however, with such observers as Meyers, Gudden, Pollak, and Haupt, claim that it is by no means a pathological lesion of insanity, but that the surroundings of the insane and the debilitated condition which so generally accompanies insanity, are the real causes for its so frequent occurrence among that class of individuals.

These observers state that in the beginning there occurs, in one or more places, a degeneration or softening of the cartilage. At these spots a gelatinous material is deposited. During this metamorphosis of tissue there are formed new blood-vessels. They arise from the perichondrium or from those vessels that supply the softened spots in the cartilage. These recently formed capillaries are comparatively of good size. Their walls are thin and abound with numerous nuclei. At the same time, then, it is noticed that while the softening of the cartilage is taking place, the perichondrium is being supplied with granulation tissue.

It has been discovered, however, that practically this same change goes on in the cartilage and perichondrium of the auricle with those who are mentally sane.

This has caused the more recent observers to contend, therefore, that hematoma auris occurs more frequently among the mentally insane, simply because as a class they are below par as regards nutrition.

The characteristic phenomenon of hematoma auris, namely, the comparatively large and rather sudden escape of blood between the cartilage and perichondrium, or into the substance of the cartilage itself, is explained by the fact that the walls of the newly formed capillaries are very thin and yield readily to pressure.

Thus it can easily be understood how a very trifling exciting cause, such as a slight blow, or even the reflex congestion following some mental emotion, could rupture one or more of these delicate vessels. On account of the pathological changes which go on in the cartilage of a typical case of hematoma auris, it has very properly been suggested, I think, that the disease under discussion be called chondromalacosis, which really indicates the essential nature of the affection.

### TREATMENT.

The treatment of hematoma auris is by no means agreed upon, nor is it, in a large class of patients, satisfactory.

Most physicians in charge of those mentally insane, even to-day, are in favor of non-interference, while aurists do not hold so generally to this idea. The tendency in this affection is to a spontaneous rupture of the blood tumor, or to a gradual absorption of the fluid. Many claim, moreover, that where the rupture occurs spontaneously, or especially by the surgeon's aid, the auricle is left in a more deformed condition than is the case when the fluid tumor is gradually absorbed. On the other hand, aurists, as a rule, assert that considerable suffering and inconvenience can be avoided and better results obtained by discriminate and intelligent interference.

I have seen some excellent results follow what we might term "the passive" as well as "the active" or surgical treatment. On the contrary, I have seen some very badly distorted and deformed auricles resulting from non-interference, which it does seem were unnecessarily in this condition.

I appreciate the fact that with the insane hematoma auris is more liable to occur; yes, nearly always does occur, in those advanced and hopeless conditions of brain disease, such as dementia and chronic melancholia; hence, the mis-shapen ear is a secondary grievance. It is natural and only fair then, I believe, among the insane, to make some allowance for not interfering with the course of the disease.

This does not, however, seem to me the proper course to pursue for those of us who at least meet this affection among the mentally sane. In the beginning of the attack it would appear proper to make use of such external applications as would afford relief to the marked tension and pain in the auricle. It is, of course, desirable to prevent as little separation of the perichondrium from the cartilage as possible, thus affording the least amount of deformity in the auricle.

Dr. Gruber, of Vienna, is in favor of an early evacuation of the effusion, and the application of pressure to insure union in the walls of the cavity which contained the blood. He uses a trocar if the blood is still fluid, but if it is coagulated he incises the tumor and removes the clot. Massage has been recommended by a few authorities, but the results do not seem to warrant such a procedure. In case incision is made, the practice of using tents to keep the wound open, at least for any length of time, should be avoided, because the secretions have in some such cases become sort of sero-purulent and chronic. Again, when incision of the tumor is practised, and it is supposed the softening of the cartilage has not ceased, it is advisable to mop out the cavity with compound tincture of iodine before applying gentle pressure. The case of hematoma auris from which the plaster-paris cast was taken, which I offer you for inspection this evening, was treated by the writer with iodine and continued pressure. The result was very satisfactory, for only the slightest deformity remained. Many phases of this subject I have purposely omitted, presenting only such as seemed most salient and suggestive.

334 Pearl Street.

